# Different associations of occupational and leisure-time physical activity with the prevalence of hypertension among middle-aged community dwellers in rural Khánh Hòa, Vietnam

**DOI:** 10.1186/s12889-023-15631-w

**Published:** 2023-04-19

**Authors:** An Dang Do, Thuy Thi Phuong Pham, Chau Que Nguyen, Dong Van Hoang, Ami Fukunaga, Shohei Yamamoto, Rachana Manandhar Shrestha, Danh Cong Phan, Masahiko Hachiya, Dong Van Huynh, Huy Xuan Le, Hung Thai Do, Tetsuya Mizoue, Yosuke Inoue

**Affiliations:** 1 Department of Community and Global Health, Graduate School of Medicine, The University of Tokyo, Tokyo, Japan; 2 Department of Non-communicable Disease Control and Nutrition, Pasteur Institute in Nha Trang, Khánh Hòa, Vietnam; 3 Department of Epidemiology and Prevention, Center for Clinical Sciences, National Center for Global Health and Medicine, Tokyo, Japan; 4 Bureau of International Health Cooperation, National Center for Global Health and Medicine, Tokyo, Japan; 5 Khánh Hòa Center for Disease Control, Khánh Hòa, Vietnam; 6 Pasteur Institute in Nha Trang, Khánh Hòa, Vietnam

**Keywords:** Occupational physical activity, Leisure-time physical activity, Hypertension, Community dwellers, Prevalence

## Abstract

**Background:**

In contrast to high-income countries where physical activity (PA), particularly leisure-time PA, has been shown to be protective against hypertension, few studies have been conducted in low- and middle-income countries. We examined the cross-sectional association between PA and hypertension prevalence among rural residents in Vietnam.

**Methods:**

We used data collected in the baseline survey of a prospective cohort study, among 3000 people aged 40–60 years old residing in rural Khánh Hòa, Vietnam. Hypertension was defined as systolic blood pressure ≥ 140 mmHg, diastolic blood pressure ≥ 90 mmHg, or the use of antihypertensive medication. We assessed occupational PA and leisure-time PA using the Global Physical Activity Questionnaire. A robust Poisson regression model was used to investigate the associations, with adjustment for covariates.

**Results:**

The prevalence of hypertension was 39.6%. After adjusting for socio-demographic and lifestyle-related variables, leisure-time PA was positively associated with hypertension prevalence (prevalence ratio [PR]: 1.03 per 10 MET-hour/week, 95% confidence interval [CI] 1.01–1.06). Occupational PA was inversely associated with hypertension prevalence (PR: 0.98 per 50 MET-hour/week, 95% CI = 0.96–0.996). After adjusting for BMI and other health-related variables, the association related to occupational PA became statistically non-significant, while the association related to leisure-time PA remained statistically significant.

**Conclusion:**

In contrast to previous studies in high-income countries, we found that leisure-time PA was positively associated with hypertension prevalence and occupational PA was associated with a lower hypertension prevalence. This suggests that the association between PA and hypertension might differ depending on the context.

**Supplementary Information:**

The online version contains supplementary material available at 10.1186/s12889-023-15631-w.

## Background

In an effort to mitigate the disease burden associated with hypertension [1], physical activity (PA), particularly leisure-time PA, has been studied extensively. It was identified as a protective factor against hypertension development [2]. For example, a meta-analysis of 29 prospective cohort studies showed that the hypertension risk was reduced by 6% (relative risk [RR]: 0.94; 95% confidence interval [CI]: 0.92–0.96) for each 10 metabolic equivalent of task [MET]-hour/week increase in leisure-time PA [3].

In contrast to the robust inverse associations observed between leisure-time PA and hypertension, findings on the association between occupational PA and hypertension have been conflicting. Meta-analyses by Huai et al. and Liu et al. did not find any evidence of a significant association [3, 4]. Moreover, some studies even reported higher risk/odds of hypertension among those who engaged in heavy occupational PA [5], while other studies have suggested that occupational PA is associated with a lower hypertension risk [6, 7].

This study was designed to extend the previous studies by examining the association between PA and the prevalence of hypertension in rural Vietnam with a special focus on the differential association between leisure-time and occupational PA. There are several reasons we believe this study is important. First, previous evidence on this topic is mostly derived from high-income countries and few studies have been conducted in low- and middle-income countries (LMICs), even though these countries have a high burden of hypertension [8, 9]. For example, in Vietnam, there was a rapid increase in the number of adults with hypertension, from 4.5 million in 1975 to 15.2 million in 2015 [10]. Second, people in LMICs, particularly those living in rural areas, might not engage in leisure-time PA as much as people in high-income countries do while they might engage in occupational PA much more than people in high-income countries. Thus, the PA–hypertension association in LMICs might not be necessarily similar to those observed in high-income countries [11, 12]. Interestingly, in a prospective cohort study in Mexico, higher moderate to vigorous PA (MVPA) at the workplace was associated with a lower hypertension risk [13], which contrasted with the observations in high-income countries.

Therefore, this study aimed to examine the association between PA and the prevalence of hypertension among middle-aged people in a rural area of Khánh Hòa province, Vietnam with a special focus on possible differences between occupational PA and leisure-time PA. We hypothesized that occupational PA is more strongly inversely associated with hypertension prevalence compared to leisure-time PA in our study setting.

## Methods

### Study setting and design

Khánh Hòa province, situated on the south-central coast of Vietnam, has a population of 1.24 million, with 57.6% of them residing in rural areas. The monthly average income per capita is 3.153 million Vietnamese dong (VND), or approximately 135 USD [14].

The Khánh Hòa Cardiovascular Study (KHCS) is an ongoing prospective cohort study that investigates the determinants of cardiovascular diseases (CVDs) in Vietnam. We selected participants from eight communes in Cam Lâm district, a rural area of Khánh Hòa province, using purposive sampling [15, 16]. The affluence level of these communes was considered average for rural Vietnam.

The baseline survey was conducted between June 2019 and June 2020. The CHC staff members created a list of 6,446 eligible individuals who were aged 40–59 years old at the time of recruitment. Of them, we invited 3,597 individuals to participate in the study until we had recruited 3000 participants (consent rate: 83.4%). We trained staff members of the Pasteur Institute in Nha Trang (PINT) with field survey experience before they collected information on lifestyle and health-related variables using a questionnaire in face-to-face interviews, and anthropometric and biochemical measurements. The detailed information of the study is available elsewhere [17, 18].

The ethical approvals were obtained at the Research Ethics Committee of the National Center for Global Health and Medicine (NCGM) (approval number: NCGM-G-003172-03) and equivalent committees at the University of Tokyo (2021007NI) and the Pasteur Institute in Nha Trang, Vietnam (02/2019/HDDD-IPN). Participants in the study provided their written consent prior to participating in the survey.

### Hypertension

Hypertension was defined as either systolic blood pressure (SBP) ≥ 140 mmHg, diastolic blood pressure (DBP) ≥ 90 mmHg, or antihypertensive medication use. Blood pressure was measured twice using an electric sphygmomanometer (Omron, HEM- 1020, Tokyo, Japan), after a 5-minute rest before the first measurement. Participants were seated with their arm supported at the heart level. The mean SBP and DBP were calculated from the two measurements.

### Physical activity

PA was assessed using the Global Physical Activity Questionnaire (GPAQ) [19–21], which was previously validated in the Vietnamese language [14] and utilized in the WHO STEPS survey in Vietnam in 2015 [22, 23]. Specifically, participants were asked about time spent doing several types of PA in a typical week. We used showcards to help participants understand the level of MVPA [24]. We calculated PA in the form of METs. Following the World Health Organization guidelines [19, 25], we defined 4 METs and 8 METs as moderate and vigorous PA, respectively. The amount of PAs was calculated as the total MET-hours/week. PAs were categorized into occupational and leisure-time activities [3].

Of the three domains that can be assessed with the GPAQ (i.e., activity at work, recreational activities, and travel to and from places), we focused on the first two domains, i.e., occupational PA (such as carrying or lifting heavy loads, digging, or construction work) and leisure-time PA (including playing sports, fitness, walking to and from places, and recreational activities).

### Covariates

Socio-demographic information was collected via the questionnaire, including age (in years), sex (male and female), education (primary school or less; secondary school; high school and higher), marital status (currently married/cohabiting or not), current employment (employed by the government/ private sector; self-employed; farmer/fisher; homemaker; others; and unemployed/retired). Information on household income was extracted from the responses by household representatives who were asked to choose a category that best described their monthly household income (VND). Values were then divided by the squared root of the number of household members to compute equivalized income, which was then categorized into tertiles (low, middle, and high). Questionnaire information was also collected on lifestyle parameters, which included smoking (never, former, and current smokers) and alcohol consumption (never, 0.1–0.9 standard drinks, 1–1.9 standard drinks, ≥ 2 standard drinks) [26].

Body mass index (BMI) was computed by dividing measured weight (kg) by the square of measured height (m^2^), which was then categorized into < 18.5; 18.5–22.9; 23.0–24.9; ≥ 25 kg/m^2^ [27]. Diabetes Mellitus (DM) was defined as either fasting plasma glucose (FPG) ≥ 126 mg/dL or hemoglobin A1c (HbA1c) ≥ 6.5% or current diabetes medication or insulin use [28]. Dyslipidemia was defined as total cholesterol ≥ 6.2 mmol/L (≥ 240 mg/dL), or low-density lipoprotein (LDL) cholesterol ≥ 4.1 mmol/L (≥ 160 mg/dL), or high-density lipoprotein (HDL) cholesterol < 1.0 mmol/L (< 40 mg/dL), or triglycerides ≥ 2.3 mmol/L (≥ 200 mg/dL), or current cholesterol-control drug use [29]. Previous diagnoses of severe diseases (i.e., diseases of the circulatory system and cancer) were also included in the models (yes or no).

### Statistical analysis

Basic characteristics are shown by leisure-time PA (with and without any PA associated with recreational activity) and occupational PA (tertiles: low, middle, and high) categories.

Multiple imputation by chained equation was used to account for missing information on household income (n = 33). Specifically, a linear regression model was used to impute household income with 200 iterations to generate 20 datasets. The estimates were combined using Rubin’s rule [30].

To investigate the association between PA and hypertension, we used a multilevel Poisson regression model with a robust variance estimator with two exposures (i.e., occupational PA and leisure-time PA) simultaneously incorporated into the model [31, 32]. We accounted for possible heterogeneity at the commune level (Level 1: individual; Level 2: commune). Three models were used in the analysis and were adjusted as follows: Model 1 was adjusted for age and sex. Model 2 was further adjusted for income, job categories, educational attainment, marital status, current smoking, and alcohol intake. Model 3 was further adjusted for health-related indicators, including BMI and physical morbidity (i.e., DM, dyslipidemia, history of cancer, or symptoms related to circulatory system diseases). Results are reported as prevalence ratios (PR) and corresponding 95% CIs of hypertension for the occupational (low, middle, and high) and the leisure-time PA categories (yes and no). We also calculated PRs per 10 MET-hour/week-increments in leisure-time PA and PRs per 50 MET-hour/week-increments in occupational PA [3].

To elucidate if a non-linear relationship existed between occupational PA and hypertension prevalence, we used a restricted cubic spline model with three knots set at the 5th, 50th, and 95th percentiles of occupational PA [33, 34] without using multiple imputation [35]. The model was adjusted for the variables included in Model 3, except for household income, which contained missing values. The covariates were rescaled based on average values before calculating the model. The limited variation in leisure-time PA reported in this study did not allow us to apply a restricted cubic spline model to leisure-time PA.

To test the robustness of the study findings, we stratified the analysis by BMI at a 23.0 kg/m^2^ cut-off, at which overweight is defined for Asian people (< 23.0; ≥ 23.0 kg/m^2^) [27], and examined whether associations differed by BMI categories (i.e., if BMI was a possible strong mediator linking PA and hypertension) [36]. We also excluded those with antihypertensive medication.

All statistical analyses were conducted using Stata ver. 15.0 (College Station, TX, USA). A restricted cubic spline plot was drawn using the package ‘mskspline2’ in Stata [34]. Statistical significance was set at p < 0.05 (two-tailed).

## Results

Table 1 shows the study participants’ basic characteristics. The mean age was 49.9 years (standard deviation [SD] = 5.5 years). Approximately 60% were female, with a higher proportion observed in those with low occupational PA. Those who attained secondary education or higher accounted for 59.5% of all participants, with a higher proportion among those with than among those without leisure-time PA (72.3% vs. 53.2%). About 30% of participants were farmers or fishers. Current smokers occupied 20.5% of all participants, with a higher proportion among those with high than among those with low occupational PA (25.0% vs. 16.4%). The prevalence of overweight/obesity tended to be higher in those with than in those without leisure-time PA (29.4% vs. 24.2%) and higher in those with low than in those with high occupational PA (28.3% vs. 23.2%). Occupational PA tended to be higher in those who did not than in those who did engage in leisure-time PA (139.2 vs. 128.8 MET-hour/week), while leisure-time PA tended to be higher in those in the lowest than in those in the highest tertile of occupational PA (7.0 vs. 5.2 MET-hour/week).

**Table 1:** Basic characteristics of study participants in the Khánh Hòa Cardiovascular Study, Vietnam (2019–2020)

Variables	Whole participants	Leisure time PA		Occupational PA		
		No	Yes	Low	Middle	High
N	3000	2000	1000	1151	900	949
Age, years (mean [SD])	49.9 [5.5]	49.6 [5.5]	50.4 [5.5]	50.4 [5.6]	50.0 [5.4]	49.2 [5.5]
Female, n (%)	1840 (61.3)	1221 (61.1)	619 (61.9)	775 (67.3)	541 (60.1)	524 (55.2)
Married/Cohabiting, n (%)	2691 (89.7)	1786 (89.3)	905 (90.5)	1021 (88.7)	814 (90.4)	856 (90.2)
Education, n (%)						
Less than primary school	352 (11.7)	279 (14.0)	73 (7.3)	118 (10.3)	116 (12.9)	118 (12.4)
Primary school	863 (28.8)	659 (33.0)	204 (20.4)	313 (27.2)	290 (32.2)	260 (27.4)
Secondary school	1068 (35.6)	725 (36.3)	343 (34.3)	375 (32.6)	311 (34.6)	382 (40.3)
High school and higher	717 (23.9)	337 (16.9)	380 (38.0)	345 (30.0)	183 (20.3)	189 (19.9)
Employment, n (%)						
Government employee	295 (9.8)	122 (6.1)	173 (17.3)	142 (12.3)	76 (8.4)	77 (8.1)
Non-Government employee	483 (16.1)	362 (18.1)	121 (12.1)	115 (10.0)	126 (14.0)	242 (25.5)
Self-employed	595 (19.8)	395 (19.8)	200 (20.0)	238 (20.7)	145 (16.1)	212 (22.3)
Farmer/Fisher	870 (29.0)	666 (33.3)	204 (20.4)	249 (21.6)	336 (37.3)	285 (30.0)
Homemaker	527 (17.6)	337 (16.9)	190 (19.0)	275 (23.9)	166 (18.4)	86 (9.1)
Others	111 (3.7)	71 (3.6)	40 (4.0)	50 (4.3)	28 (3.1)	33 (3.5)
Unemployed/retired	119 (4.0)	47 (2.4)	72 (7.2)	82 (7.1)	23 (2.6)	14 (1.5)
Equivalized Income, n (%)						
Low	1002 (33.4)	722 (36.1)	280 (28.0)	380 (33.0)	295 (32.8)	327 (34.5)
Middle	1045 (34.8)	719 (36.0)	326 (32.6)	390 (33.9)	319 (35.4)	336 (35.4)
High	920 (30.7)	536 (26.8)	384 (38.4)	372 (32.3)	272 (30.2)	276 (29.1)
Missing	33 (1.1)	23 (1.2)	10 (1.0)	9 (0.8)	14 (1.6)	10 (1.1)
Smoking, n (%)						
Never smoking	2036 (67.9)	1350 (67.5)	686 (68.6)	844 (73.3)	604 (67.1)	588 (62.0)
Former smoking	350 (11.7)	196 (9.8)	154 (15.4)	118 (10.2)	108 (12.0)	124 (13.1)
Current smoking	614 (20.5)	454 (22.7)	160 (16.0)	189 (16.4)	188 (20.9)	237 (25.0)
Alcohol consumption (standard drink), n (%)						
0	2114 (70.5)	1431 (71.6)	683 (68.3)	865 (75.2)	637 (70.8)	612 (64.5)
0.1–0.9	416 (13.9)	253 (12.7)	163 (16.3)	128 (11.1)	126 (14.0)	162 (17.1)
1-1.9	201 (6.7)	125 (6.3)	76 (7.6)	69 (6.0)	59 (6.6)	73 (7.7)
≥2	269 (9.0)	191 (9.6)	78 (7.8)	89 (7.7)	78 (8.7)	102 (10.7)
Body mass index (kg/m^2^), n (%)						
<18.5	139 (4.6)	104 (5.2)	35 (3.5)	52 (4.5)	47 (5.2)	40 (4.2)
18.5–22.9	1344 (44.5)	949 (47.5)	395 (39.5)	476 (41.4)	403 (44.8)	465 (49.0)
23.0-24.9	739 (24.6)	463 (23.2)	276 (27.6)	297 (25.8)	218 (24.2)	224 (23.6)
≥25.0	778 (25.9)	484 (24.2)	294 (29.4)	326 (28.3)	232 (25.8)	220 (23.2)
Total occupational MET-hour/week [SD]	135.7 [110.7]	139.2 [114.3]	128.8 [102.8]	31.4 [33.3]	134.1 [25.0]	263.7 [85.0]
Total leisure-time MET-hour/week [SD]	6.1 [13.7]	0 [0]	18.2 [18.6]	7.0 [15.2]	5.8 [11.2]	5.2 [13.9]
Systolic blood pressure, mean [SD]	130.9 [19.1]	130.6 [19.1]	131.6 [19.1]	131.5 [18.9]	131.0 [19.9]	130.1 [18.5]
Diastolic blood pressure, mean [SD]	83.9 [12.1]	83.8 [12.2]	84.1 [12.0]	84.1 [12.1]	84.5 [12.3]	83.9 [12.0]
Antihypertensive medication, n (%)	367 (12.2)	203 (10.2)	164 (16.4)	168 (14.6)	107 (11.9)	92 (9.7)
Diabetes	307 (10.2)	189 (9.5)	118 (11.8)	140 (12.2)	90 (10.0)	77 (8.1)
Dyslipidemia	1352 (45.1)	865 (43.3)	487 (48.7)	532 (46.2)	423 (47.0)	397 (41.8)
Previous diagnosis of serious diseases (cancer or diseases of circulatory system), n (%)	175 (5.8)	102 (5.1)	73 (7.3)	82 (7.1)	44 (4.9)	49 (5.2)

PA: Physical activity; SD: Standard Deviance; CVD: Cardiovascular Diseases; MET: Metabolic Equivalent by Task

Leisure-time PA range: 0–0 (no); 0.67–280 (yes); Occupational PA range: 0–84 (Low); 88–168 (Middle); 172.7–896 (High)

Of the study participants, 39.6% had hypertension. Table 2 shows the results of the multilevel robust Poisson models examining the association between PA and hypertension prevalence. After adjusting for age and sex, those in the highest occupational PA category were less likely to be hypertensive than those in the lowest category (PR 0.89, 95% CI 0.77–1.02), while the PR per 50 MET-hour/week-increment in occupational PA was 0.98 (95% CI 0.96–0.997). Leisure-time PA was positively associated with hypertension prevalence (PR 1.12, 95% CI 1.03–1.22 for those with vs. those without leisure-time PA; PR 1.04 per 10 MET-hour/week-increment, 95% CI 1.01–1.06). These associations were slightly attenuated but did not change markedly when the models were adjusted for educational attainment, household income, job categories, marital status, current smoking, and alcohol intake in Model 2, and for BMI and physical morbidity in Model 3.

**Table 2:** Results of multilevel Poisson regression with a robust variance estimator examining the association between occupational and leisure-time physical activity and hypertension among study participants of the Khánh Hòa Cardiovascular Study in Vietnam (2019–2020)

	Model 1			Model 2			Model 3	
	PR	95%CI		PR	95%CI		PR	95%CI
Occupational PA								
Categorical								
Low	1.00	Ref.		1.00	Ref.		1.00	Ref.
Middle	0.89	0.79–1.01		0.90	0.80–1.01		0.91	0.81–1.02
High	0.89	0.77–1.02		0.89	0.78–1.03		0.92	0.81–1.05
Per 50 MET hour/week	0.98	0.96–0.997		0.98	0.96–0.996		0.98	0.97–1.001
Leisure-time PA								
Categorical								
No	1.00	Ref.		1.00	Ref.		1.00	Ref.
Yes	1.12	1.03–1.22		1.13	1.05–1.22		1.07	1.00–1.15
Per 10 MET hour/week	1.04	1.01–1.06		1.03	1.01–1.06		1.02	1.003–1.04

Model 1 was adjusted for age and sex. Model 2 was further adjusted for educational attainment, marital status, employment, income, smoking status, and alcohol consumption. Model 3 was adjusted for variables included in Model 2 plus body mass index and co-morbidity (i.e., dyslipidemia, diabetes, and previous history of serious diseases). We accounted for clustering by study communes (n = 8) by employing a multilevel model (Level 1: individuals; Level 2: study communes)

**Fig. 1:**
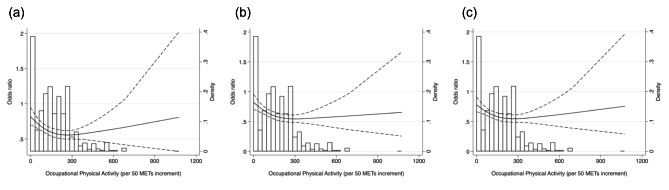
Restricted cubic spline model of odds ratio of hypertension prevalence with Occupational Physical activity (per 50 METs increment) Panel **(a)** represents results of restricted cubic spline model adjusting for age, sex and leisure-time PA (Model 1); Panel **(b)**shows results of restricted cubic spline model further adjusting for educational attainment, marital status, employment, smoking status, and alcohol consumption (Model 2); Panel **(c)** represents results of restricted cubic spline model further adjusting for body mass index and co-morbidity (i.e., dyslipidemia, diabetes and previous history of serious diseases) (Model 3).

Figure 1 illustrates the non-linear relationship between occupational PA and hypertension prevalence based on the cubic spline model. The OR of hypertension constantly decreased until occupational PA of 300 MET-hour/week, where the OR reached the minimum value, and increased among those with occupational PA at the upper end of the distribution. The coefficients obtained from the models are shown in Supplementary Table 1.

When we stratified the participants by BMI categories, significant positive associations related to leisure-time PA were found among individuals with BMI < 23.0 kg/m^2^ (Model 2: PR 1.03, 95% CI 1.01–1.06), but not among those with BMI ≥ 23.0 kg/m^2^ (Table 3). On the other hand, significant inverse associations in relation to occupational PA were observed among those with BMI ≥ 23 kg/m^2^ (Model 2: PR 0.97, 95% CI 0.93–0.998), while we did not find evidence of a significant association among those with BMI < 23.0 kg/m^2^.

**Table 3:** Results of Poisson regression with a robust variance estimator examining the association between occupational and leisure-time physical activity and hypertension among study participants of the Khánh Hòa Cardiovascular Study in Vietnam (2019–2020), stratified by BMI categories (< 23 kg/m^2^; ≥23 kg/m^2^)

	Model 1			Model 2			Model 3	
	PR	95%CI		PR	95%CI		PR	95%CI
**BMI < 23 (n = 1483)**								
Occupational PA per 50 MET hour/week	0.997	0.96–1.04		0.99	0.95–1.04		1.00	0.96–1.04
Leisure-time PA per 10 MET hour/week	1.03	1.00-1.06		1.03	1.01–1.06		1.03	1.01–1.05
**BMI ≥ 23 (n = 1517)**								
Occupational PA per 50 MET hour/week	0.97	0.94–0.999		0.97	0.93–0.996		0.97	0.94–0.997
Leisure-time PA per 10 MET hour/week	1.02	0.98–1.06		1.02	0.99–1.05		1.01	0.98–1.05

Model 1 was adjusted for age and sex. Model 2 was further adjusted for educational attainment, marital status, employment, income, smoking status, and alcohol consumption. Model 3 was adjusted for variables included in Model 2 plus body mass index and co-morbidity (i.e., dyslipidemia, diabetes, and previous history of serious diseases). Study communes (n = 8) were treated as clusters in the statistical models

After excluding those on antihypertensive medication in the sensitivity analysis, we found a significant inverse association with occupational PA, but no evidence of a significant association with leisure-time PA (Supplementary Table 2).

## Discussion

Among 3000 rural residents in Khánh Hòa province, Vietnam, we found a positive association between leisure-time PA and hypertension prevalence, while occupational PA was associated with a lower hypertension prevalence. More specifically, every 10 MET-hour/week increment in leisure-time PA was associated with a 2–4% increase in hypertension prevalence, while every 50 MET-hour/week-increment in occupational PA was linked to a 2% reduction in hypertension prevalence. These associations did not materially change after adjusting for socioeconomic status and lifestyle-related variables. Our hypothesis that occupational PA is more strongly inversely associated with hypertension prevalence than leisure-time PA was supported.

The inverse association between occupational PA and hypertension prevalence observed in our study was not consistent with the results of a meta-analysis of 13 cohort studies, the majority of which were conducted in high-income countries, by Huai et al. [4], which did not find any evidence of a significant association between occupational PA and hypertension (RR: 0.93 in high- vs. low-occupational PA groups, 95% CI: 0.81–1.08). However, our finding was in line with several studies conducted in LMICs that reported inverse associations between occupational PA and hypertension [13, 37–39]. For example, in an 8-year prospective cohort study among 27 provinces and three megacities in China, by Gu et al., the RR for hypertension was 1.27 (95% CI: 1.10–1.47) among those with low vs. those with high occupational PA [37].

There are some possible interpretations for these discrepant findings between high-income countries and LMICs. First, compared to high-income countries, where a higher occupational PA often indicates a lower socio-economic status (SES), it is possible that there was no such linkage in LMICs, particularly in rural communities where a fair proportion of the population engaged in physically demanding jobs (e.g., farming and fishery), irrespective of their SES [40]. Second, the absolute amount and duration of occupational PA may be larger and longer, respectively, in Vietnam and other LMICs than in high-income countries [41]. In fact, the mean occupational PA in this study population was 135.7 MET-hour/week (SD 110.7 MET-hour/week). This was markedly higher than that in European countries, for example, 47.7 MET-hour/week in Germany [41]. In countries where people do not engage in much occupational PA, it is theoretically not possible to observe the beneficial effects of work-related exercise on blood pressure (e.g., a decrease in vascular resistance caused by decreased sympathetic activity, improving insulin sensitivity, lowering adiposity, and improving energy balance).

Our finding that leisure-time PA was linked to a higher prevalence of hypertension was not in line with the findings of the meta-analyses by Huai et al. and Liu et al. [3, 4] or those of some more recent studies [38, 42]. For example, the pooled RR of hypertension among high vs. low leisure-time PA was 0.81 (95% CI 0.76–0.85) [4]. Our participants lived in rural communities in Vietnam, where people are engaged in agriculture, unstable work, and seasonal jobs [14, 23, 43], and do not typically engage in leisure-time PA. Only a small proportion of the study participants (33.3%) engaged in leisure-time PA and the absolute amount of leisure-time PA was also limited. It is also possible that differences in basic characteristics between those who did and those who did not engage in leisure-time PA differed so markedly that it was not possible to adjust for them fully in a regression model (i.e., residual confounding). For example, those who engaged in leisure-time PA tended to be richer, more educated, and have higher excess body weight than those who did not engage in leisure-time PA.

The restricted cubic spline model applied in this study suggested that a declining trend in the prevalence of hypertension reversed into an increasing trend when occupational PA exceeded 300 MET-hour/week, albeit with large confidence intervals towards the upper end of occupational PA. Given that most participants had occupational PA below this level, we concluded that occupational PA was inversely associated with the prevalence of hypertension. However, this reversal in the association might indicate a possible detrimental effect of occupational PA on hypertension. This suggestive U-shaped association between occupational PA and hypertension agrees with a previous study conducted in China, which found a U-shaped association between occupational PA and the incidence of hypertension [39].

When we stratified participants by BMI at a cut-off of 23.0 kg/m^2^, we found the inverse association between occupational PA and hypertension only among those with BMI ≥ 23.0 kg/m^2^. Thus, the protective effect of occupational PA seemed to be more significant among those with obesity. This could be interpreted that, compared to those with BMI < 23.0 kg/m^2^ who were metabolically healthy irrespective of their occupational PA level, those with higher BMI may develop better cardiorespiratory fitness via occupational PA, which might protect them from developing hypertension [44, 45]. On the other hand, the positive association between leisure-time PA and the prevalence of hypertension was more pronounced among those with BMI < 23 kg/m^2^. While this suggests that occupational PA and leisure-time PA have different health implications, depending on the BMI of the individuals, it should be considered that this association might have been due to residual confounding [36].

An inconsistency in the direction of the associations between different types of PA and health outcomes within a single study had been reported previously (i.e., the PA paradox). More specifically, occupational PA increased, and leisure-time PA reduced the risk of various negative health outcomes. Our study also reported inconsistency in these associations, but the pattern of inconsistency was not the same as those reported previously (inverse association of hypertension with occupational PA and no association with leisure-time PA).

This study had several limitations. First, as occupational PA and leisure-time PA were both measured using a questionnaire, the responses might have been affected by recall bias. More specifically, using the GPAQ to assess PA could lead to an overestimate or underestimate of PA. For example, it is possible that engaging in housework or taking care of babies were not considered occupational physical activity by rural Vietnamese middle-aged people. Second, the cross-sectional design of the current study could not establish any causal inference. For example, it is possible that those who had developed hypertension chose to engage in leisure-time PA and avoid occupational PA. However, it should also be mentioned that results in relation to occupational PA did not change markedly when we excluded those on antihypertensive medication in our sensitivity analysis. Third, our study participants were not representative of the Vietnamese population as they resided in rural communities of a single province and were all middle-aged. Thus, caution should be exercised when generalizing the study findings to other populations or age groups.

Future research should (1) examine whether the associations observed in this study can also be obtained in a longitudinal design and (2) subjectively measure PA by using accelerometers to reduce misclassification bias in relation to the exposure. In addition, similar studies conducted in other locations (e.g., urban areas of Vietnam or rural areas of other LMICs) will facilitate our understanding of the association between PA and hypertension in a global context.

## Conclusion

Among 3000 rural residents in Khánh Hòa Province, Vietnam, we found that a higher level of occupational PA was associated with a lower hypertension prevalence while leisure-time PA was positively associated with hypertension. These findings contrasted with those of previous studies conducted in high-income countries, suggesting that the associations between PA and hypertension might differ depending on the context. Public health researchers and practitioners working in LMICs should be aware of the possible difference in the association between PA and hypertension in such countries.

## Electronic supplementary material

Below is the link to the electronic supplementary material.

Supplementary Material 1

## Data Availability

The data are not publicly available but are available upon reasonable request to the corresponding author (yosuke.yoshi.yosky@gmail.com).

## Abbreviations

BMI: Body mass index
CI: Confidence intervals
DBP: Diastolic blood pressure
DM: Diabetes mellitus
FPG: Fasting plasma glucose
GPAQ: Global Physical Activity Questionnaire
HbA1c: Hemoglobin A1c
HDL: High-density lipoprotein
IQR: Interquartile range
KHCS: Khánh Hòa Cardiovascular Study
LDL: Low-density lipoprotein
LMICs: Low- and middle-income countries
METs: Total metabolic equivalent task
MICE: Multiple imputation by chained equation
MVPA: Moderate to vigorous physical activity
NCGM: National Center for Global Health and Medicine
OR: Odds ratio
PA: Physical activity
PINT: Pasteur Institute in Nha Trang
PR: Prevalence ratio
SD: Standard deviation
SES: Socio-economic status
SBP: Systolic blood pressure
USA: The United State of America
USD: United States dollar
WHO: World Health Organization
