# Supplementary material for: Different associations of occupational and leisure-time physical activity with the prevalence of hypertension among middle-aged community dwellers in rural Khánh Hòa, Vietnam
Source: BMC Public Health. 2023 Apr 19;23:713. doi: 10.1186/s12889-023-15631-w (PMC10116664; doi:10.1186/s12889-023-15631-w)
Supplement: Supplementary file 1 — Supplementary Material 1 [file 12889_2023_15631_MOESM1_ESM.pdf]

Supplementary table 1. The parameters of the curvilinear relationship between occupational physical activity and hypertension prevalence.

OPA	coef.	Stand error	95%CI Lower	95%CI Upper
Model 1				
OPA 50th percentile	-0.154	0.045	-0.243	-0.065
OPA 95th percentile	0.184	0.072	0.042	0.327
Model 2				
OPA 50th percentile	-0.154	0.046	-0.244	-0.064
OPA 95th percentile	0.170	0.073	0.025	0.314
Model 3				
OPA 50th percentile	-0.140	0.048	-0.233	-0.047
OPA 95th percentile	0.166	0.076	0.018	0.318

Model 1 was adjusted for age and sex. Model 2 was further adjusted for educational attainment, marital status, smoking status, and alcohol consumption. Model 3 was adjusted for variables included in Model 2 plus body mass index and co-morbidity (i.e., dyslipidemia, diabetes and previous history of serious diseases). Study communes (n = 8) were treated as clusters in the statistical models.

Supplementary table 2. Results of Poisson regression with a robust variance estimator examining the association between occupational and leisure-time physical activity and hypertension among study participants of the Khánh Hòa Cardiovascular Study in Vietnam (2019-2020), excluding those under antihypertensive medication (n = 2,633).

	**Model 1**		**Model 2**		**Model 3**	
	PR	95%CI	PR	95%CI	PR	95%CI
Occupational PA						
Categorical						
Low	1.00	Ref.	1.00	Ref.	1.00	Ref.
Middle	0.89	0.75-1.07	0.88	0.75-1.03	0.88	0.74-1.03
High	0.90	0.77-1.06	0.89	0.77-1.02	0.91	0.79-1.04
Per 50 MET hour/week	0.98	0.96-0.997	0.98	0.96-0.99	0.98	0.96-0.996
Leisure-time PA						
Categorical						
No	1.00	Ref.	1.00	Ref.	1.00	Ref.
Yes	1.03	0.93-1.14	1.04	0.94-1.15	0.99	0.91-1.09
Per 10 MET hour/week	1.01	0.97-1.06	1.01	0.97-1.05	1.00	0.96-1.04

Model 1 was adjusted for age and sex. Model 2 was further adjusted for educational attainment, marital status, employment, income, smoking status, and alcohol consumption. Model 3 was adjusted for variables included in Model 2 plus body mass index and co-morbidity (i.e., dyslipidemia, diabetes, and previous history of serious diseases). Study communes (n = 8) were treated as clusters in the statistical models.
